# Influence of corticosteroid treatment on CXCR4 expression in DLBCL

**DOI:** 10.1186/s13550-023-00993-4

**Published:** 2023-05-10

**Authors:** Sebastian Martin, David Viertl, Anna Janz, Stefan Habringer, Ulrich Keller, Margret Schottelius

**Affiliations:** 1 Translational Radiopharmaceutical Sciences, Department of Nuclear Medicine and Department of Oncology, Centre Hospitalier Universitaire Vaudois (CHUV), University of Lausanne, Rue du Bugnon 25A, Agora, CH-1011 Lausanne, Switzerland; 2 AGORA, Pôle de Recherche Sur Le Cancer, 1011 Lausanne, Switzerland; 3 SCCL Swiss Cancer Center Leman, 1011 Lausanne, Switzerland; 4 PentixaPharm GmbH, 97080 Würzburg, Germany; 5 Department of Hematology, Oncology and Cancer Immunology, Campus Benjamin Franklin, Charité - Universitätsmedizin Berlin, corporate member of Freie Universität Berlin and Humboldt-Universität zu Berlin, Berlin, Germany; 6 German Cancer Consortium (DKTK), German Cancer Research Center (DKFZ), Heidelberg, Germany; 7 Max Delbrück Center (MDC), 13092 Berlin, Germany

**Keywords:** CXCR4, Chemokine receptor regulation, Cortisol, Corticosteroids, PentixaTher, Radioligand therapy

## Abstract

**Background:**

CXCR4-targeted radioligand therapy (RLT) with [^177^Lu]Lu/[^90^Y]Y-PentixaTher has recently evolved as a promising therapeutic option for patients with advanced hematological cancers. Given their advanced disease stage, most patients scheduled for PentixaTher RLT require concomitant or bridging chemotherapy to prevent intermittent tumor progression. These (mostly combination) therapies may cause significant downregulation of tumoral CXCR4 expression, challenging the applicability of PentixaTher RLT. This study therefore aimed at investigating the influence of corticosteroids, a central component of these chemotherapies, on CXCR4 regulation in diffuse large B cell lymphoma (DLBCL).

**Methods:**

Different DLBCL cell lines (Daudi, OCI-LY1, SUDHL-4, -5-, -6 and -8) as well as the human T-cell lymphoma cell line Jurkat were incubated with Dexamethasone (Dex; 0.5 and 5 µM, respectively) and Prednisolone (Pred; 5 and 50 µM, respectively) for different time points (2 h, 24 h). Treatment-induced modulation of cellular CXCR4 surface expression was assessed via flow cytometry (FC) and compared to untreated cells. A radioligand binding assay with [^125^I]CPCR4.3 was performed in parallel using the same cells. To quantify potential corticosteroid treatment effects on tumoral CXCR4 expression in vivo, OCI-LY1 bearing NSG mice were injected 50 µg Dex/mouse i.p. (daily for 6 days). Then, a biodistribution study (1 h p.i.) using [^68^Ga]PentixaTher was performed, and tracer biodistribution in treated (n = 5) vs untreated mice (n = 5) was compared.

**Results:**

In the in vitro experiments, a strongly cell line-dependent upregulation of CXCR4 was observed for both Dex and Pred treatment, with negligible differences between the high and low dose. While in Jurkat, Daudi and SUDHL-8 cells, CXCR4 expression remained unchanged, a 1.5- to 3.5-fold increase in CXCR4 cell surface expression was observed for SUDHL-5 < SUDHL-4 /-6 < OCI-LY1 via FC compared to untreated cells. This increase in CXCR4 expression was also reflected in correspondingly enhanced [^125^I]CPCR4.3 accumulation in treated cells, with a linear correlation between FC and radioligand binding data. In vivo, Dex treatment led to a general increase of [^68^Ga]PentixaTher uptake in all organs compared to untreated animals, as a result of a higher tracer concentration in blood. However, we observed an overproportionally enhanced [^68^Ga]PentixaTher uptake in the OCI-LY1 tumors in treated (21.0 ± 5.5%iD/g) vs untreated (9.2 ± 2.8%iD/g) mice, resulting in higher tumor-to-background ratios in the treatment group.

**Conclusion:**

Overall, corticosteroid treatment (Dex/Pred) consistently induced an upregulation of CXCR4 expression DBLCL cells in vitro, albeit in a very cell line-dependent manner. For the cell line with the most pronounced Dex-induced CXCR4 upregulation, OCI-LY1, the in vitro findings were corroborated by an in vivo biodistribution study. This confirms that at least the corticosteroid component of stabilizing chemotherapy regimens in DLBCL patients prior to [^177^Lu]Lu-PentixaTher RLT does not lead to downregulation of the molecular target CXCR4 and may even have a beneficiary effect. However, further studies are needed to investigate if and to what extent the other commonly used chemotherapeutic agents affect CXCR4 expression on DLBCL to ensure the choice of an appropriate treatment regimen prior to [^177^Lu]Lu/[^90^Y]Y-PentixaTher RLT.

## Background and study design

CXCR4-targeted radioligand therapy (RLT) with [^177^Lu]Lu/[^90^Y]Y-PentixaTher (Yttrium (^90^Y) anditixafortide) has recently evolved as a promising therapeutic option for patients with advanced hematological cancers such as multiple myeloma (MM), diffuse large B cell lymphoma (DLBCL) or acute lymphoblastic leukemia (ALL) [1–3]. Generally, a prerequisite for patient eligibility for [^177^Lu]Lu/[^90^Y]Y-PentixaTher RLT is high tumoral CXCR4 expression, confirmed by high uptake of the companion diagnostic, [^68^Ga]Ga-PentixaFor (Gallium (^68^Ga) boclatixafortide), in the respective tumor lesions by pre-therapeutic PET/CT. However, as most patients scheduled for PentixaTher RLT suffer from advanced stages of their disease, concomitant or bridging chemotherapy to prevent tumor progression between diagnostic imaging and RLT is often required. That these intermittent therapeutic regimens may have significant impact on tumoral CXCR4 expression has recently been demonstrated. For three patients with different hematological malignancies, substantial downregulation of CXCR4 expression in response to bridging chemotherapy was observed [4], rendering [^177^Lu]Lu/[^90^Y]Y-PentixaTher RLT unsuitable or possibly much less effective.

To date, the mechanisms of this CXCR4 downregulation are unclear, and the observed effects are all the more surprising since two of the reported patients received Dexamethasone (albeit in conjunction with cyclophosphamide or other chemotherapeutic agents [4]). Dexamethasone was shown to substantially increase surface expression of CXCR4 in MM cells [4, 5], in murine B-cells [6] and in human T-cells [7]. In view of the implementation of an early phase clinical study on [^177^Lu]Lu/[^90^Y]PentixaTher RLT in patients with DLBCL, it is of particular importance to understand the role of corticosteroid treatment on the regulation of CXCR4 expression in this malignancy. Such information could possibly guide the safe use of bridging chemotherapies prior to considering the patients for CXCR4-targeted [^177^Lu]Lu/[^90^Y]Y-PentixaTher RLT.

Thus, this study aimed at investigating the influence of corticosteroid (Dexamethasone, Prednisolone) treatment on CXCR4 expression in a panel of DLBCL cell lines (Daudi, OCI-LY1, SUDHL-4, SUDHL-5, SUDHL-6, SUDHL-8) with different baseline CXCR4 expression levels. The human T-cell leukemia cell line Jurkat with high CXCR4 expression was also included.

Clinically, in the management of DLBCL, the combination of CHOP (Cyclophosphamide, Doxorubicin, Vincristine and Prednisone^a^) and rituximab is considered a standard first-line treatment [8], whereas modified/extended DHAP (Dexamethasone, Cytarabine, Cisplatin) protocols are used as second-line chemotherapies [9]. Since patients with DLBCL eligible for [^177^Lu]Lu/[^90^Y]Y-PentixaTher RLT are very likely to undergo/have undergone one of these treatments, Prednisolone^1^ and Dexamethasone were both included into this investigation.

To ensure the reliability of the in vitro data, the concentrations for Dexamethasone and Prednisolone were chosen such as to resemble as closely as possible to the maximum plasma concentrations observed in humans receiving standard treatments (i.e., 40 mg Dexamethasone/day [9] and 100 mg Prednisone/day [8]). On the basis of this dosing, the corresponding maximum plasma concentrations for Dexamethasone and Prednisolone were found to be approximately 0.5 µM [10] and 5 µM [11], respectively. Thus, these concentrations were used consistently throughout the study. In some experiments, however, to assess a potential concentration dependence of the (up)regulation of CXCR4 and of cell viability, a tenfold concentration of the chosen corticosteroids was also investigated.

Data from the literature indicate variable kinetics of CXCR4 upregulation in different cell types, with the first detection of receptor upregulation ranging from 1 to 3 h [6, 7] to 24 h of incubation [5]. We therefore performed initial pilot studies to establish the most suitable incubation time for detecting potential effects of corticosteroid treatment on CXCR4 expression in the different DLBCL cell lines. Ultimately, after having established appropriate experimental conditions, a second set of experiments was performed, in which the changes in CXCR4 expression observed in flow cytometry were correlated with changes in radioligand binding induced by corticosteroid therapy. Given the particularly high sensitivity of [^125^I]CPCR4.3 for quantifying different CXCR4 expression levels in vitro [12], this ligand was used instead of [^177^Lu]Lu-PentixaTher for the in vitro studies.

## Results

### Pilot studies—time dependence and concentration dependence

**Fig. 1:**
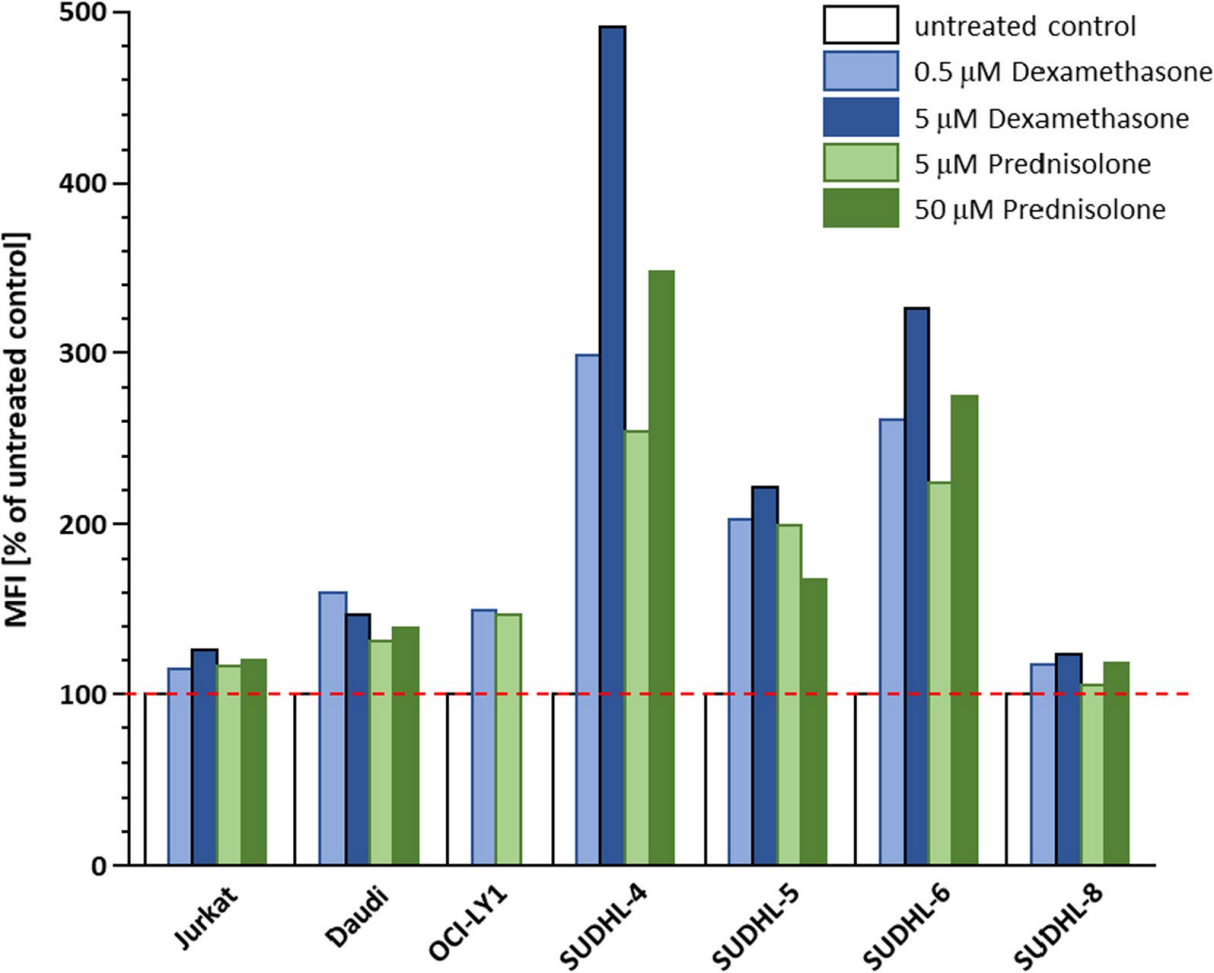
Dependence of CXCR4 upregulation on drug concentration. Data are shown as the mean fluorescence intensity observed by flow cytometry for each of the cell lines at different concentrations (low and high dose) of Dexamethasone and Prednisolone, respectively, after 24 h of incubation in percent of untreated controls (n = 1)

In a first set of experiments, the time dependence of CXCR4 (up)regulation by corticosteroid treatment was investigated. As opposed to results from the literature [6, 7], an incubation time of 2 h at 37 °C with both the high dose (5 µM Dexamethasone, 50 µM Prednisolone) and the respective low-dose mimicking plasma concentration (0.5 µM Dexamethasone, 5 µM Prednisolone) did not induce any notable change in CXCR4 expression in any of the cell lines investigated (data not shown). However, after 24 h of incubation, flow cytometry analysis revealed increased CXCR4 surface expression levels for Daudi, OCI-LY1, SUDHL-4, SUDHL-5 and SUDHL-6 cells at both drug concentrations (Fig. 1). Consequently, an incubation time of 24 h was selected for all subsequent experiments.

Importantly, the effect of corticosteroid treatment on CXCR4 surface expression was highly variable between cell lines (Fig. 1). For SUDHL-4, SUDHL-5 and SUDHL-6 cells, substantial CXCR4 upregulation by treatment with Dexamethasone and Prednisolone was observed, which was also found to be dependent on drug concentration in the case of SUDHL-4 and SUDHL-6 cells. Only moderate, concentration-independent CXCR4 upregulation was observed for Daudi and OCI-LY1 cells, whereas the treatment effect was negligible for Jurkat and SUDHL-8 cells. Overall, there was a slight trend toward a more notable CXCR4 upregulation by Dexamethasone than by Prednisolone in the responding cell lines (Daudi, OCI-LY1, SUDHL-4, -5 and -6). However, since these pilot experiments were performed only once for estimation of effects (n = 1), their significance is limited and does not allow conclusive interpretation. Despite the observed variability between different cell lines in response to corticosteroid treatment, however, no negative effect of Dexamethasone and Prednisolone on CXCR4 surface expression was detected.

### Correlation of treatment effects observed by flow cytometry with radioligand binding data

Based on the above pilot experiments, OCI-LY1, SUDHL-4 and SUDHL-5 cells were selected for more in-depth evaluation of the association of steroid pretreatment, CXCR4 expression and CXCR4 radioligand uptake, based on their gradual response to corticosteroid treatment (SUDHL-4 > SUDHL-5 > OCI-LY1). Despite pronounced CXCR4 upregulation by corticosteroid treatment, SUDHL-6 cells were not included due to practical considerations.

**Fig. 2:**
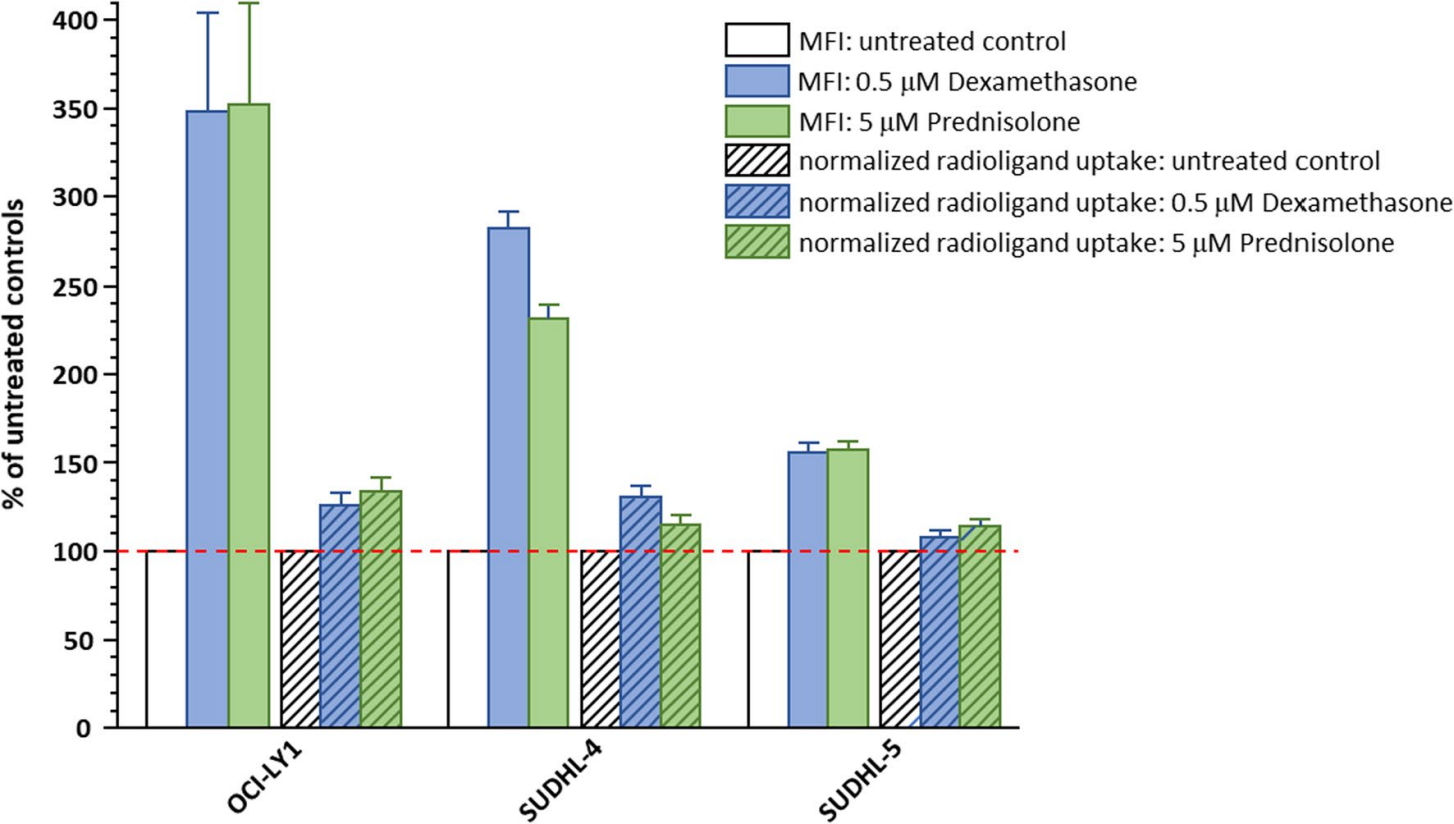
CXCR4 upregulation by corticosteroid treatment as assessed in parallel by flow cytometry and a radioligand binding study using [^125^I]CPCR4.3. All data are shown in percent of the values obtained for untreated control cells. Flow cytometry data are shown as relative mean fluorescence intensity (MFI) values and are means ± SD from 3–4 separate determinations with n = 3, respectively. Radioligand binding data are shown as relative normalized uptake values (% of added dose bound per 1 Mio live cells) and are means ± SD from 2 separate determinations with n = 3, respectively

To be able to assess the influence of the CXCR4 upregulation observed via flow cytometry on radioligand uptake an additional set of experiments was performed. Aliquots of the same treated cells (24 h, 37 °C) were analyzed in parallel via flow cytometry and via incubation with [^125^I]CPCR4.3 to quantify CXCR4 expression. Results are summarized in Fig. 2.

**Fig. 3:**
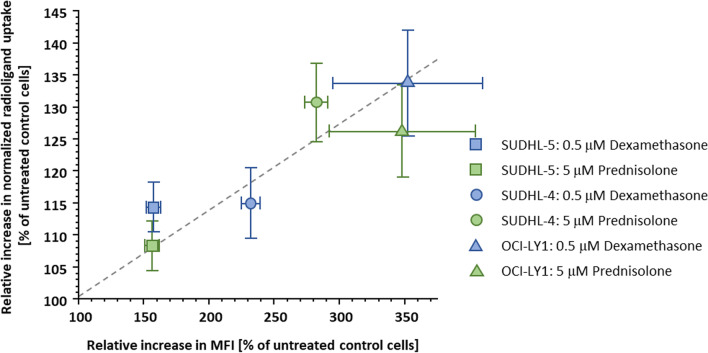
Correlation of CXCR4 upregulation by corticosteroid treatment quantified by flow cytometry and via a radioligand binding study, respectively. All data are shown in percent of the values obtained for untreated control cells. Flow cytometry data are shown as relative MFI values and are means ± SD from 3–4 separate determinations with n = 3, respectively. Radioligand binding data using [^125^I]CPCR4.3 are shown as relative normalized uptake values (% of added dose bound per 1 Mio live cells) and are means ± SD from 2 separate determinations with n = 3, respectively

Interestingly, the extent of CXCR4 upregulation observed by flow cytometry for the three selected cell lines was quite different from the pilot experiments, with the responsiveness to therapy being now in the order of OCI-LY1 > SUDHL-4 > SUDHL-5. Another unexpected finding is the fact that the increase in cellular uptake of [^125^I]CPCR4.3 was much less pronounced than the change in CXCR4 surface expression observed by flow cytometry. However, as shown in Fig. 3, there is a linear correlation between the relative changes in CXCR4 expression determined by flow cytometry and via [^125^I]CPCR4.3 binding. This correlation on the one hand corroborates the initial observation (see pilot experiments), that corticosteroid treatment does increase CXCR4 expression in DLBCL cell lines. However, the extent of this effect is strongly cell line dependent. The observed increase in radioligand uptake in cell lines with a strong CXCR4 upregulation upon corticosteroid treatment may even prove beneficial in the context of RLT with [^177^Lu]Lu/[^90^Y]Y-PentixaTher.

### In vivo assessment of the effect of Dexamethasone treatment on tumoral CXCR4 expression

To verify this hypothesis, mice bearing subcutaneous OCI-LY1 DLBCL xenografts were randomized into a control group (no treatment) and a treatment group (50 μg Dexamethasone i.p. for 6 consecutive days), and a comparative biodistribution study using [^68^Ga]Ga-PentixaTher was carried out at the end of the treatment period. Of note, since at the time of the experiment, ^177^LuCl_3_ was not available from the manufacturer due to production shortages, ^68^Ga-labeled PentixaTher was chosen as a substitute for the therapeutic agent [^177^Lu]Lu-PentixaTher.

**Fig. 4:**
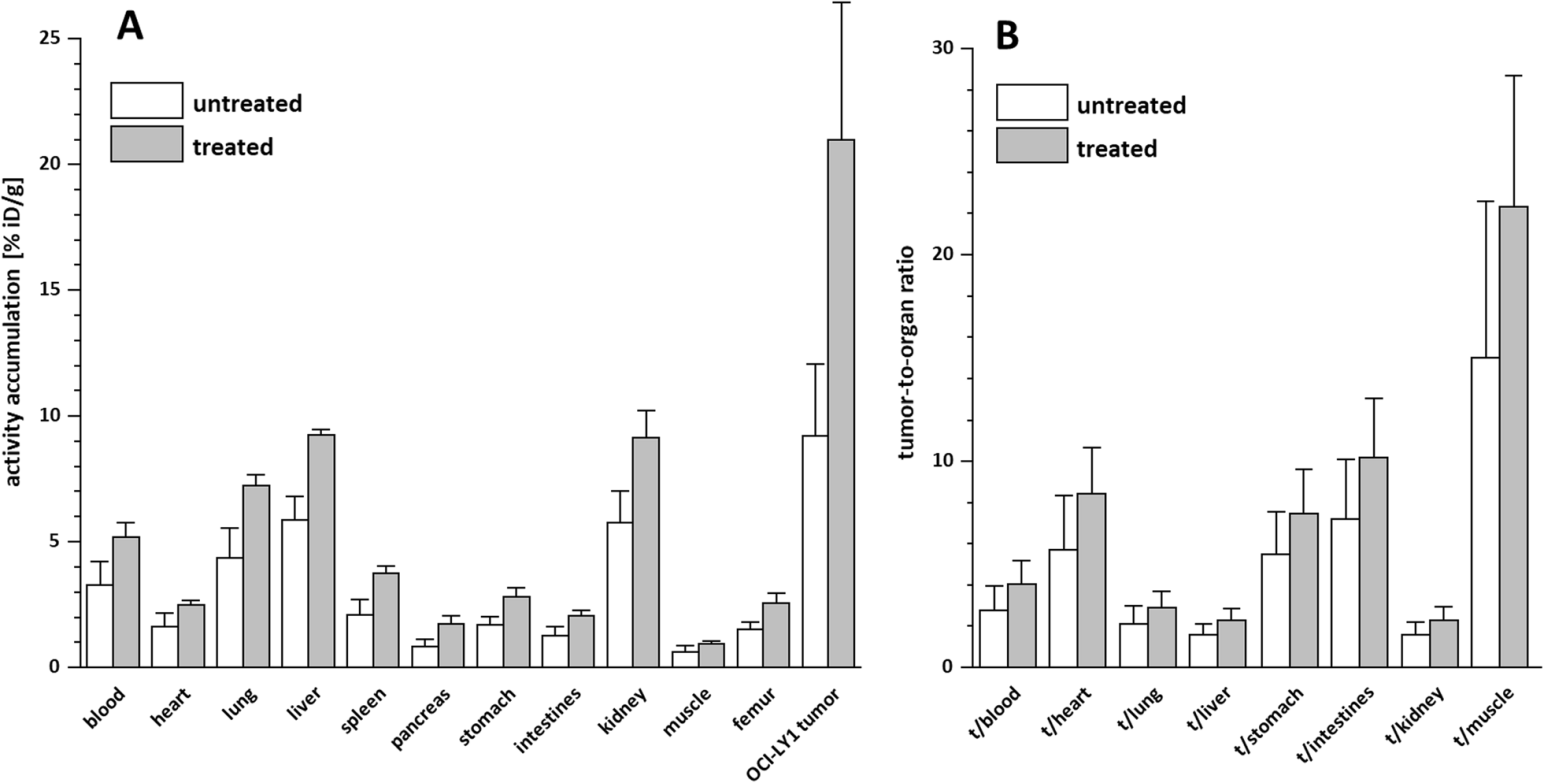
Biodistribution (**A**) and Tumor-to-organ ratios (**B**) of [^68^Ga]Ga-PentixaTher in OCI-LY1 DLBCL xenograft bearing NSG mice at 1 h p.i.. The treatment group (n = 5) received 50 μg Dexamethasone i.p. for 6 consecutive days before tracer injection. The biodistribution data are given in % injected dose per gram tissue (%iD/g) and are means ± SD (n = 5 animals/group)

As summarized in Fig. 4, Dexamethasone treatment (dosing as in [6]) had a pronounced effect on the overall biodistribution of [^68^Ga]Ga-PentixaTher. The observed increased concentration of the tracer in blood (increase by 58% in treated vs untreated animals) was reflected by a 53–68% higher absolute tracer uptake in all organs in the treated animals. The only exception from this consistent general effect was the OCI-LY1 xenograft, with an increase in [^68^Ga]Ga-PentixaTher uptake by 128% compared to untreated animals. As of now, the reasons for the tendency toward an increased blood concentration of [^68^Ga]Ga-PentixaTher are unclear; it may either be the result of a delayed blood clearance as a side effect of corticosteroid treatment, or be related to an upregulation of mCXCR4 expression on circulating mouse immune cells (T-lymphocytes, B-cells [6]). [^177^Lu]Lu-PentixaTher is known to display moderate affinity toward mCXCR4 [13], and it is highly probable that the mCXCR4 affinity of [^68^Ga]Ga-PentixaTher lies in the same range, and thus, the increased [^68^Ga]Ga-PentixaTher concentration in blood in treated animals may thus be related to specific tracer binding. However, further experiments are needed to confirm this hypothesis.

In contrast, however, the over-proportional increase in tracer accumulation in the OCI-LY1 xenograft is in line with our in vitro findings, i.e., a dexamethasone-treatment induced CXCR4 upregulation on the tumor cells. This is further underlined by the consistently higher tumor/organ ratios observed for the treated animals (Fig. 4). Although the differences in tumor/background ratios between the treated and the untreated animals are not statistically significant (P = 0.4–0.8 for all organs) due to the relatively high standard deviation of the absolute tumor uptake value for the treatment group, our data nevertheless indicate a clear trend, which is in accordance with our in vitro observations.

In summary, we observed that corticosteroid treatment (Dexamethasone, Prednisolone) consistently induced an upregulation of CXCR4 expression DBLCL cells in vitro. Of note, the effect varied significantly between cell lines, the increase ranging from 20 to 300% of baseline CXCR4 expression. For the cell line with the most pronounced response to Dexamethasone treatment, OCI-LY1, the in vitro findings could also be recapitulated in the corresponding in vivo xenograft model. This confirms that at least the corticosteroid component of stabilizing chemotherapy regimens in DLBCL patients [8, 9] prior to CXCR4-targeted RLT with [^177^Lu]Lu-PentixaTher does not lead to downregulation of the molecular target CXCR4 and may even have a contrary, beneficiary effect. However, it needs to be investigated in more detail to which extent rituximab or the other chemotherapeutic agents used in CHOP or DHAP treatment protocols affect CXCR4 expression, since these effects may limit the use of CXCR4-targeted diagnostics and/or CXCR4-targeted therapies [4]. A better understanding of CXCR4 (de)regulation by DLBCL lymphoma directed chemotherapies may help to ensure the choice of an appropriate treatment regimen prior to [^177^Lu]Lu/[^90^Y]Y-PentixaTher RLT in these diseases.

## Materials and methods

### Cell culture

Jurkat human T-cell leukemia cells were cultured in RPMI-1640 medium, supplemented with 10% FCS. All DLBCL cell lines, namely Daudi, OCI-LY1, SUDHL-4, -5, -6 and -8, were kindly supplied by Prof. Ulrich Keller, Department of Hematology and Oncology, Charité, Berlin, Germany, and were grown in RPMI-1640 medium, supplemented with 20% FCS. All cell lines were maintained at 37 °C in a humidified 5% CO_2_ atmosphere. Media and supplements were obtained from Biochrom (Berlin, Germany) or Gibco (life technologies, Darmstadt, Germany). For cell counting, an automated CytoSMART Lux cell counter (Axion BioSystems, Atlanta, USA) was used.

For treatment with Dexamethasone and Prednisolone (obtained as suspensions/solutions for oral application from the clinical pharmacy at CHUV), the respective cell suspensions were centrifuged (3 min, 1300 rcf, Megafuge 1.0, Heraeus Thermo Scientific). The culture medium was removed and the cell pellet was resuspended in assay medium (DMEM/F-12 medium with Glutamax-I (1:1) supplemented with 5% BSA) to yield a cell suspension with a concentration of app. 5–7·10^6^ cells/ml. For treatment, either 140 μL of assay medium (untreated control cells) or 140 μL of tenfold concentrated solutions of Dexamethasone and Prednisolone (5 μM and 50 μM as well as 50 μM and 500 μM, respectively) was added to 1.26 mL of cell suspension. After incubation of the cells at 37° for 24 h in an incubator, the cells were centrifuged, washed once with assay medium, and resuspended in assay medium to a concentration of 5·10^6^ cells/mL. This suspension was either used directly for the radioligand binding assay or processed further for flow cytometry analysis.

### Flow cytometry

The treated and untreated cells were washed twice with cold flow cytometry buffer (5% fetal bovine serum in PBS). For the staining, triplicates of 1·10^6^ cells were prepared and incubated 45 min on ice with a concentration of 1 µg/mL PE anti-human CD184 CXCR4 antibody (BioLegend) or PE mouse IgG2a isotype control (BioLegend) in 100 µL FACS buffer. Next, the cells were spun down at 300 × g and the staining agent was discarded. The cells were washed twice and were resuspended in 500 µL of cold FACS buffer. In addition, DAPI was added to each sample shortly before the analysis to yield a final concentration of 0.5 µg/mL. The flow cytometry analyses were conducted immediately on a Beckman CoulterGallios flow cytometer. The acquired data were analyzed with FlowJo v10.7.1.

### Radioligand binding assay

Radioiodination of CXCR4.3 was carried out using the IodoGen® method as described previously [12].

For the binding assay, samples containing app. 1·10^6^ cells in assay medium were incubated with [^125^I]CPCR4.3 (0.2 nM) at RT for 60 min in the presence (non-specific binding) or absence (control) of 100 µM unlabeled CPCR4.3 (n = 3 per condition, total sample volume: 250 µL). After incubation, the tubes were centrifuged (3 min, 1300 rcf, Megafuge 1.0, Heraeus Thermo Scientific) and the supernatant was carefully removed. The cells were washed once with 200 µL of cold HBSS, and the supernatant of the washing step was pooled with the supernatant from the previous step (free ligand). Then, the amount of bound radioligand in the cell pellet as well as the amount of free radioligand in the combined supernatants was quantified using a γ-counter (WALLAC; 1480 WIZARD™ 3″). For each sample, the cellular uptake in % of total added dose was calculated and then used for further data processing.

### Tumor model and in vivo biodistribution studies

For induction of tumor growth, female NSG mice (6–8 weeks) were subcutaneously injected with 5 × 10^6^ OCI-LY1 cells in HBSS/Matrigel (1:1). After 25 days, small palpable tumors had grown in all animals, and animals were divided into a control group (no treatment, n = 5) and a treatment group (n = 5). Treated animals received 50 μg Dexamethasone in 100 μL PBS as an i.p. injection for 6 consecutive days (day 25-day 30 post tumor implant). The following day, all animals were injected intravenously with 3–4 MBq (0.16–0.18 nmol) [^68^Ga]Ga-PentixaTher, and a biodistribution study was carried out. The animals were sacrificed at 1 h post injection (p.i.), and the organs of interest were dissected. The radioactivity was measured in weighted tissue samples using a γ-counter. Data are expressed in % ID/g tissue (mean ± SD).

## Data Availability

All data generated or analyzed during this study are included in this published article.

## Footnotes

1 Prednisolone is the actual active compound; Prednisone (administered in CHOP) is the prodrug for Prednisolone and is metabolized to Prednisolone in the liver.
